# Update on leukodystrophies and developing trials

**DOI:** 10.1007/s00415-023-11996-5

**Published:** 2023-09-27

**Authors:** Giorgia Ceravolo, Kristina Zhelcheska, Violetta Squadrito, David Pellerin, Eloisa Gitto, Louise Hartley, Henry Houlden

**Affiliations:** 1 Department of Neuromuscular Disorders, Institute of Neurology, University College London (UCL), London, UK; 2 Department of Clinical and Experimental Medicine, University of Messina, Messina, Italy; 3 Neonatal and Paediatric Intensive Care Unit, Department of Clinical and Experimental Medicine, University of Messina, Messina, Italy; 4 Royal London Hospital, Barts NHS, London, UK

**Keywords:** Leukodystrophy, Genetic testing, Trials

## Abstract

Leukodystrophies are a heterogeneous group of rare genetic disorders primarily affecting the white matter of the central nervous system. These conditions can present a diagnostic challenge, requiring a comprehensive approach that combines clinical evaluation, neuroimaging, metabolic testing, and genetic testing. While MRI is the main tool for diagnosis, advances in molecular diagnostics, particularly whole-exome sequencing, have significantly improved the diagnostic yield. Timely and accurate diagnosis is crucial to guide symptomatic treatment and assess eligibility to participate in clinical trials. Despite no specific cure being available for most leukodystrophies, gene therapy is emerging as a potential treatment avenue, rapidly advancing the therapeutic prospects in leukodystrophies. This review will explore diagnostic and therapeutic strategies for leukodystrophies, with particular emphasis on new trials.

**Supplementary Information:**

The online version contains supplementary material available at 10.1007/s00415-023-11996-5.

## Introduction

Leukodystrophies comprise a heterogeneous group of rare genetic disorders that primarily affect the white matter of the central nervous system (CNS) [1, 2]. In a broader context, leukoencephalopathy is a comprehensive term used to describe all white matter disorders, which can stem from a variety of causes, including genetic factors [3].

Previous studies conducted in the USA and Europe reported an incidence of leukodystrophies, ranging from 1 in 100,000 to 1 in 6000–7700 live births, while Asian countries report an incidence of 3 per 100,000 live births [4–6]. Metachromatic leukodystrophy and X-linked adrenoleukodystrophy have been reported to be the most common leukodystrophies [7, 8].

**Fig. 1:**
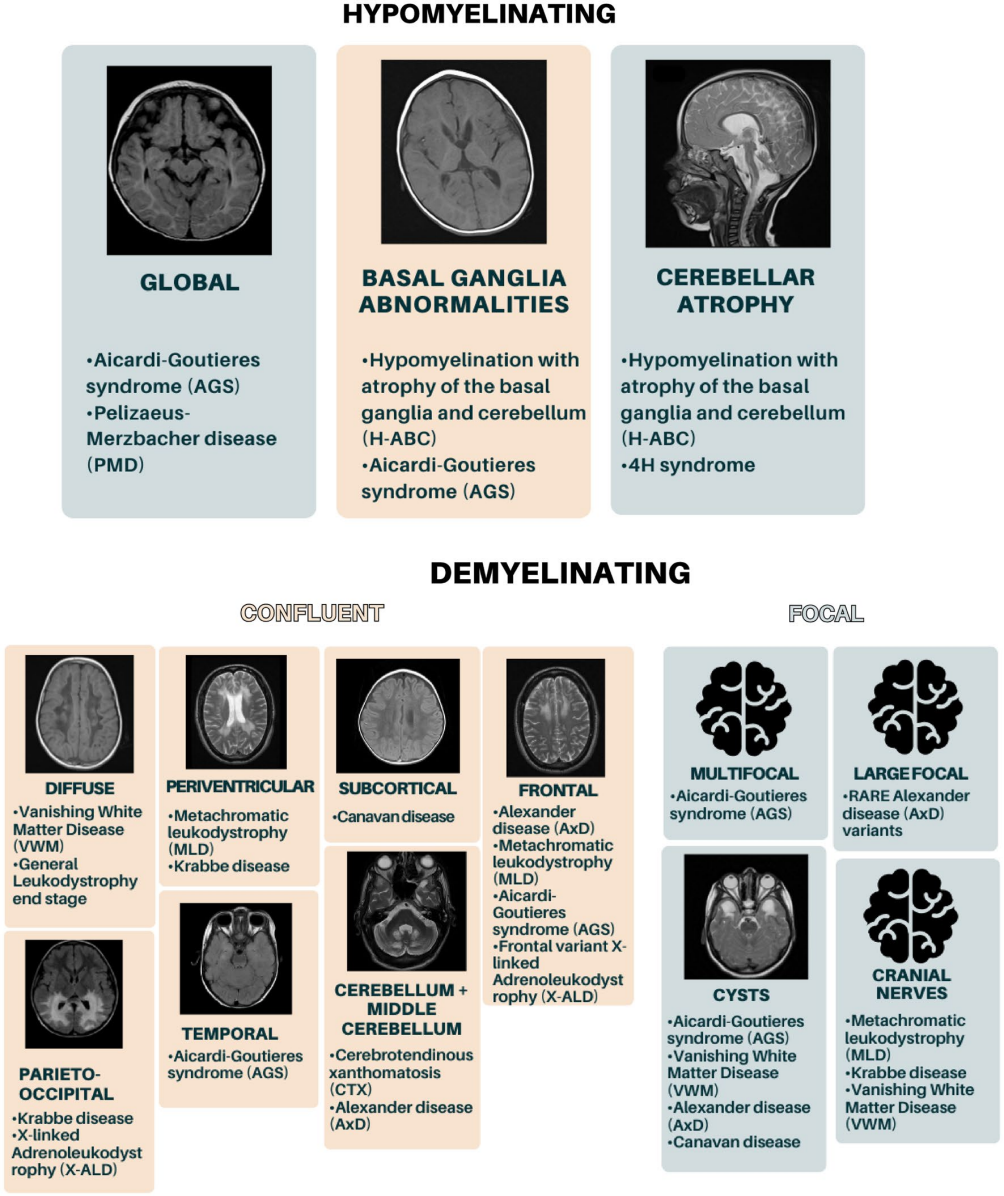
Classification of leukodystrophies in hypomyelinating and demyelinating forms [11–19]

**Fig. 2:**
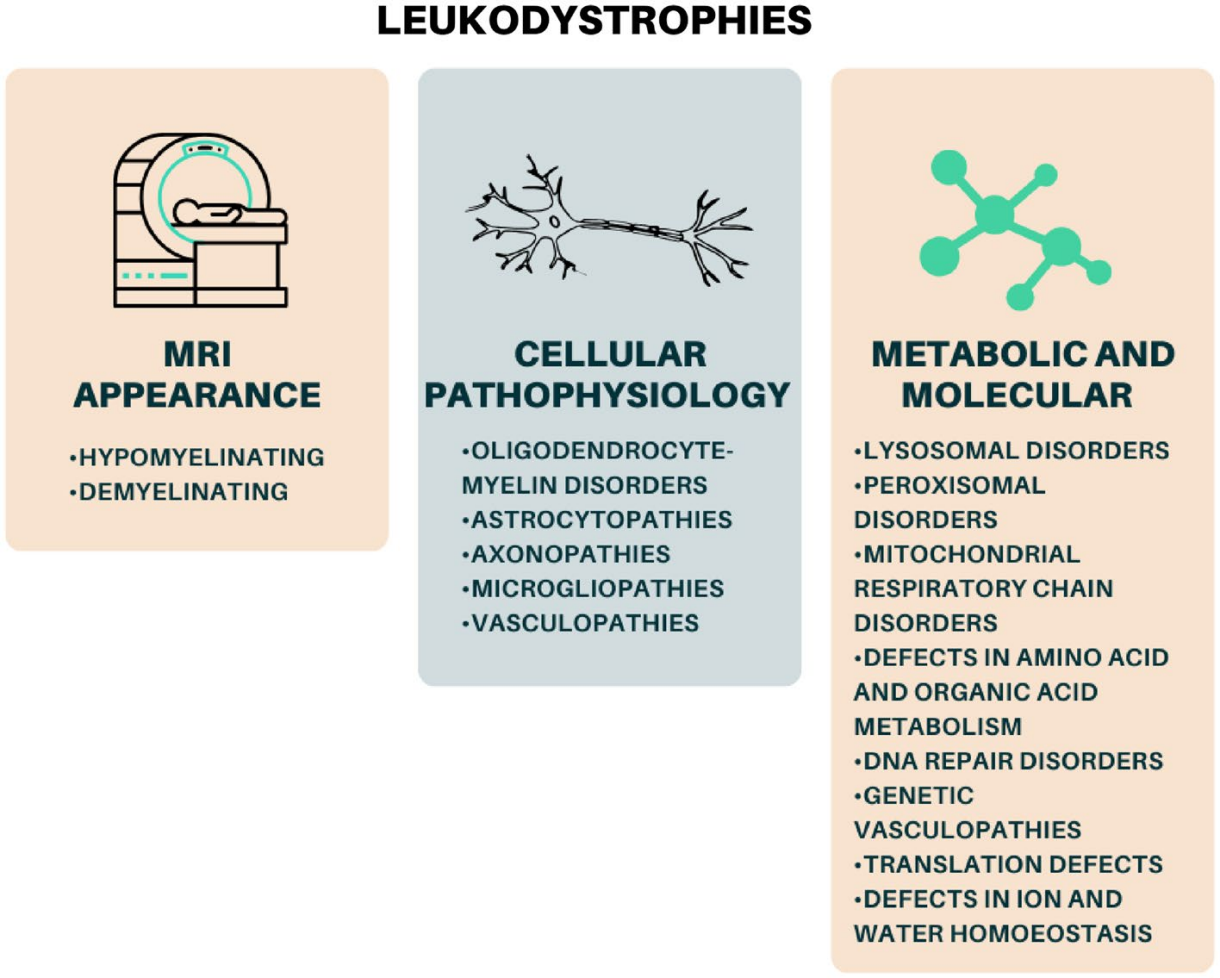
Classification of leukodystrophies based on MRI appearance, cellular pathophysiology, and metabolic and molecular mechanisms

Leukodystrophies can be broadly classified into demyelinating and hypomyelinating types based on their appearance on magnetic resonance imaging (MRI) (Fig. 1), while alternative categorizations are based on cellular pathophysiology and metabolic and molecular approaches [3, 9, 10] (Fig. 2). MRI and genetic testing are crucial for confirming the diagnosis of leukodystrophy. Molecular testing is pursued when a diagnosis is suspected, and thanks to the use of whole-exome sequencing (WES) and whole-genome sequencing (WGS), the diagnostic yield has rapidly increased in recent years.

Leukodystrophies can be inherited in various manners including autosomal recessive (AR), autosomal dominant (AD), X-linked (XL), additionally, both recessive and dominant inheritance, sporadic forms, and mitochondrial inheritance (Supplementary Material Table 1). The most common inheritance is as an AR condition. Here, we will provide an overview of the clinical and genetic aspects of leukodystrophies and present an update on current trials.

### Pathophysiology

At the cellular level, due to the crucial role of the different CNS cells in generating and supporting the integrity of myelin, leukodystrophies are not only caused by defects in the myelin but also by dysfunction in other CNS structures [3, 9]. Similarly, at the molecular level, disruptions in one pathway can have far-reaching consequences on other pathways (Table 1) [10]. An in-depth understanding of the pathomechanisms underlying leukodystrophies is crucial for the development of innovative therapies.

**Table 1:** The role of different CNS structures and molecular pathways in the pathophysiology of leukodystrophies

	Mechanism	Examples of leukodystrophies
*Cellular level*		
Oligodendrocyte-myelin defects	Oligodendrocytes play a vital role in the production and maintenance of myelin, which is crucial for efficient signal transmission in the CNS. They are involved in de novo myelination, remodelling existing myelin, and facilitating remyelination after injury. Additionally, oligodendrocytes provide metabolic support necessary for the health and functioning of myelin structures	PMD, MLD, Krabbe disease, Canavan disease, X-ALD
Astrocytes defects	Astrocytes play a crucial role in the development of the brain and CNS homeostasis. They support the blood–brain barrier, regulate ion–water homeostasis, ensure synaptic plasticity, promote neurite outgrowth, and aid in repair after injury. Disruptions in astrocyte functions can lead to myelin damage and intra-myelin oedema	MLC, ODDD, AGS, AxD, VWM
Axons	Axons initiate myelin generation, influence myelination levels, and promote axonal and myelin integrity through bi-directional signalling. Axonal signals also regulate myelin thickness, reflecting neuronal activity and ensuring proper function	POLR3, 4H
Microglia	Microglia, as the primary innate immune cells in the CNS, play a crucial role in maintaining homeostasis and regulating myelin maintenance. They possess the capability to either promote or hinder the differentiation of oligodendrocyte precursor cells, thereby influencing remyelination. Additionally, microglia contribute to the clearance of myelin debris following CNS injury, which is vital for effective repair mechanisms	ALSP, LCC
Small blood vessels	Injuries to small arteries, arterioles, venules, and capillaries resulting from insufficient oxygen supply. These injuries frequently lead to the formation of white matter lesions, lacunar infarcts, and haemorrhages	Retinal vasculopathy with cerebral leukodystrophy (RVCL-S)
*Molecular level*		
Lysosomal disorders [20]	Genetic disorders characterized by impaired lysosomal function, leading to the accumulation of undegraded macromolecules within cells	Krabbe disease, MLD
Peroxisomal disorders [21]	Peroxisomes are essential organelles involved in various metabolic pathways such as fatty acid β-oxidation, synthesis of plasmalogens, and redox homeostasis. When the peroxisomal function is impaired, can result in structural and functional abnormalities in the white matter	X-ALD
Mitochondrial respiratory chain disorders [22, 23]	The mitochondrial respiratory chain is responsible for producing ATP, the energy currency of cells. It plays a vital role in cellular metabolism and is also involved in the formation of larger complexes with diverse functions. Variations in the components of this chain can lead to mitochondrial diseases with various clinical manifestations	LBSL, LTBL
Disorders of amino acid and organic acid metabolism [24]	Inborn errors involving the breakdown of amino acids and organic acids. Each specific disorder corresponds to a particular metabolic pathway and can impact brain development by depleting essential metabolites or causing the accumulation of toxic intermediates	Canavan disease
DNA repair disorders [25]	DNA repair disorders disrupt the maintenance of genomic integrity, leading to a higher susceptibility to DNA damage and genomic instability. Impaired DNA repair can stall RNA polymerase transcription, resulting in transcription blocks and accumulation of DNA damage	Cockayne syndrome
Genetic vasculopathies	Genetic vasculopathies encompass a group of disorders affecting the blood vessels due to genetic mutations. Each specific vasculopathy has its unique mechanisms and clinical manifestations	AGS
Translation defects [26]	Translation defects lead to disrupting protein production. While protein synthesis is essential for cellular function, the diverse range of disease phenotypes observed suggests a remarkable heterogeneity in the manifestations of these disorders	VWM, 4H leukodystrophy, LBSL, LTBL
Defects in ion and water homoeostasis [27, 28]	The proper balance of ions and water is essential for the electrical activity and functioning of neurons in the brain. Disruptions in ion and water homeostasis, caused by defects in ion channels, transporters, or regulatory mechanisms, can lead to cellular and physiological dysfunctions, potentially resulting in severe brain damage or death	MLC
Errors of intermediary metabolism [29]	Intermediary metabolism refers to the complex highly integrated network of biochemical reactions involved in energy production, reducing power and synthesis of biomolecules. This integrated metabolic network ensures the proper functioning and survival of cells	Canavan disease, CTX

*ALSP* adult-onset leukoencephalopathy with axonal spheroids and pigmented glia, *AGS* Aicardi–Goutieres syndrome, *AxD* Alexander disease, *CTX* cerebrotendinous xanthomatosis, *LTBL* hypomyelination with brainstem and leukoencephalopathy with thalamus and brainstem involvement and high lactate, *LCC* leukodystrophy with calcifications and cyst, *LBSL* leukoencephalopathy with brainstem and spinal cord involvement and lactate elevation, *MLC* megalencephalic leukoencephalopathy with subcortical cysts, *MLC* megalencephalic leukoencephalopathy with subcortical cysts, *MLD* metachromatic leukodystrophy, *MLD* metachromatic leukodystrophy, *ODDD* oculodentodigital dysplasia, *PMD* Pelizaeus–Merzbacher disease, *POLR3* POLR3-related disorders, *VWM* vanishing white matter disease, *X-ALD* X-linked adrenoleukodystrophy

### Clinical manifestations

Clinical manifestations of leukodystrophies vary depending on the specific aetiology and age of onset, which can range from birth to adulthood. However, there is a spectrum of disease presentation across all age groups, with symptoms and signs changing accordingly (Table 2). Hypomyelinating diseases tend to present earlier than demyelinating forms and early age of onset is usually associated with more severe manifestations. Leukodystrophies are progressive disorders, although some rare cases show significant improvements over time, both clinically and radiologically [9, 30]. Differentiating neurological and non-neurological symptoms can be helpful in suspecting a specific diagnosis.

**Table 2:** Common non-neurological manifestations in leukodystrophies

Ophthalmological findings	Neonatal VWM, HCC Cataract Congenital Later in life Nystagmus Congenital Later in life Retinal glistening white dots Retinal vascular defect Optic atrophy Glaucoma Myopia Microphthalmia/+ persistent pupillary membrane, iris abnormalities, and microcornea	Neonatal VWM, HCC CTX, 18q-syndrome, Cockayne Several hypomyelinating Canavan, PMD, PMLD POLR3, 4H, ODDD, 18q- SLS (pathognomonic) CRMCC, RVCL-S Canavan, CTX, VWM, mitochondrial disorders AGS POLR3 Cockayne/ODDD
Endocrinological findings	Adrenocortical insufficiency Hypogonadotropic hypogonadism Hypothyroidism GH deficiency Ovarian failure	X-ALD, POLR3 POLR3, 4H, X-ALD, HLD7 18q-, POLR3, 4H, AGS 18q-, POLR3, 4H VWM
Dental findings	Hypodontia, oligodontia Malocclusion	POLR3, 4H, HLD7 ODDD
Cutaneous findings	Ichthyosis Chilblains Hyperpigmentations Cutaneous photosensitivity Tendinous xanthoma	SLS, MSD AGS X-ALD, AMN Cockayne syndrome CTX
Musculoskeletal findings	Syndactyly Dysostosis multiplex Osteoporosis Scoliosis and dislocations of large joints	ODDD MSD, MPS CTX HLD26
Gastrointestinal findings	Diarrhoea Frequent vomiting Gallbladder disease Hepatosplenomegaly	CTX, early-onset peroxisomal disorders AxD MLD AGS, MSD, early-onset peroxisomal disorders
Cardiological findings	Cardiomegaly	18q-
Additional findings	Symptoms of congenital infections Deafness	AGS HLD23

*18q-* 18q deletion syndrome, *4H* 4H syndrome,AGS Aicardi–Goutieres syndrome, *CRMCC* cerebroretinal microangiopathy with calcifications and cysts, *CTX* cerebrotendinous xanthomatosis, *HLD23* hypomyelinating leukodystrophy-23 with ataxia, deafness, liver dysfunction, and dilated cardiomyopathy, *HLD26* hypomyelinating leukodystrophy-26 with chondrodysplasia, *HLD7* hypomyelinating leukodystrophy-7 with or without oligodontia and/or hypogonadotropic hypogonadism, *MPS* mucopolysaccharidosis, *MSD* multiple sulfatase deficiency, *ODDD* oculodentodigital dysplasia, *PMD* Pelizaeus–Merzbacher disease, *POLR3* POLR3-related disorders, RVCL-S retinal vasculopathy with cerebral leukodystrophy, *SLS* Sjögren–Larsson syndrome, *VWM* vanishing white matter disease *X-ALD* X-linked adrenoleukodystrophy

*Non-neurological symptoms* can involve different systems and can be typical of a specific type of leukodystrophy (Table 2).

#### Neurological manifestations

The neurological signs and symptoms observed in leukodystrophies are diverse and provide valuable insights into the underlying conditions. Hypomyelinating forms are characterized by delayed motor milestone acquisition, while demyelinating forms tend to show motor regression.

The most common symptoms are motor and cognitive difficulties. Pyramidal symptoms tend to be symmetrical starting in the lower extremities and leading to spasticity and limited ambulatory abilities. Extrapyramidal movement disorders, such as dystonia, are commonly found in leukodystrophies [hypomyelinating leukodystrophy-6 (HABC)]; seizures can either be early symptoms [Alexander disease (AxD [31]), vanishing white matter disease (VWM [32])] or may occur in later stages. Gait abnormalities can present as an isolated slowly progressive ataxia [metachromatic leukodystrophy (MLD [33])] or more often associated with peripheral sensory neuropathy [leukodystrophy with calcifications and cyst (LCC), leukoencephalopathy with brainstem and spinal cord involvement and lactate elevation (LBSL [34])]. Peripheral neuropathy can be one of the first manifestations in some leukodystrophies such as Krabbe disease [35] and it is also usually present in cerebrotendinous xanthomatosis (CTX [36]), Pelizaeus–Merzbacher disease (PMD [37]), and Pelizaeus–Merzbacher-like disease (PMLD). Bulbar symptoms can often occur along with other neurological findings (MLD [33], X-linked adrenoleukodystrophy (X-ALD), Canavan disease [38], and AxD [3]). Neurobehavioural abnormalities, such as irritability [Krabbe disease [35], Aicardi–Goutieres syndrome (AGS [39])], attention deficit (X-ALD [40]), academic difficulties, anxiety, and depression, are common in leukodystrophies. Macrocephaly (AxD) and microcephaly (AGS [39], HLD17, HLD12) can narrow down the diagnosis. Leukodystrophies such as MLD [33], Krabbe disease [35], and X-ALD [40] have all been associated with additional neurologic features, such as autonomic dysfunction.

Recognition of the neurological manifestations can provide insights into the underlying condition and further assist in refining the diagnosis (Supplementary Material Table 1).

### Diagnostic workflow

**Fig. 3:**
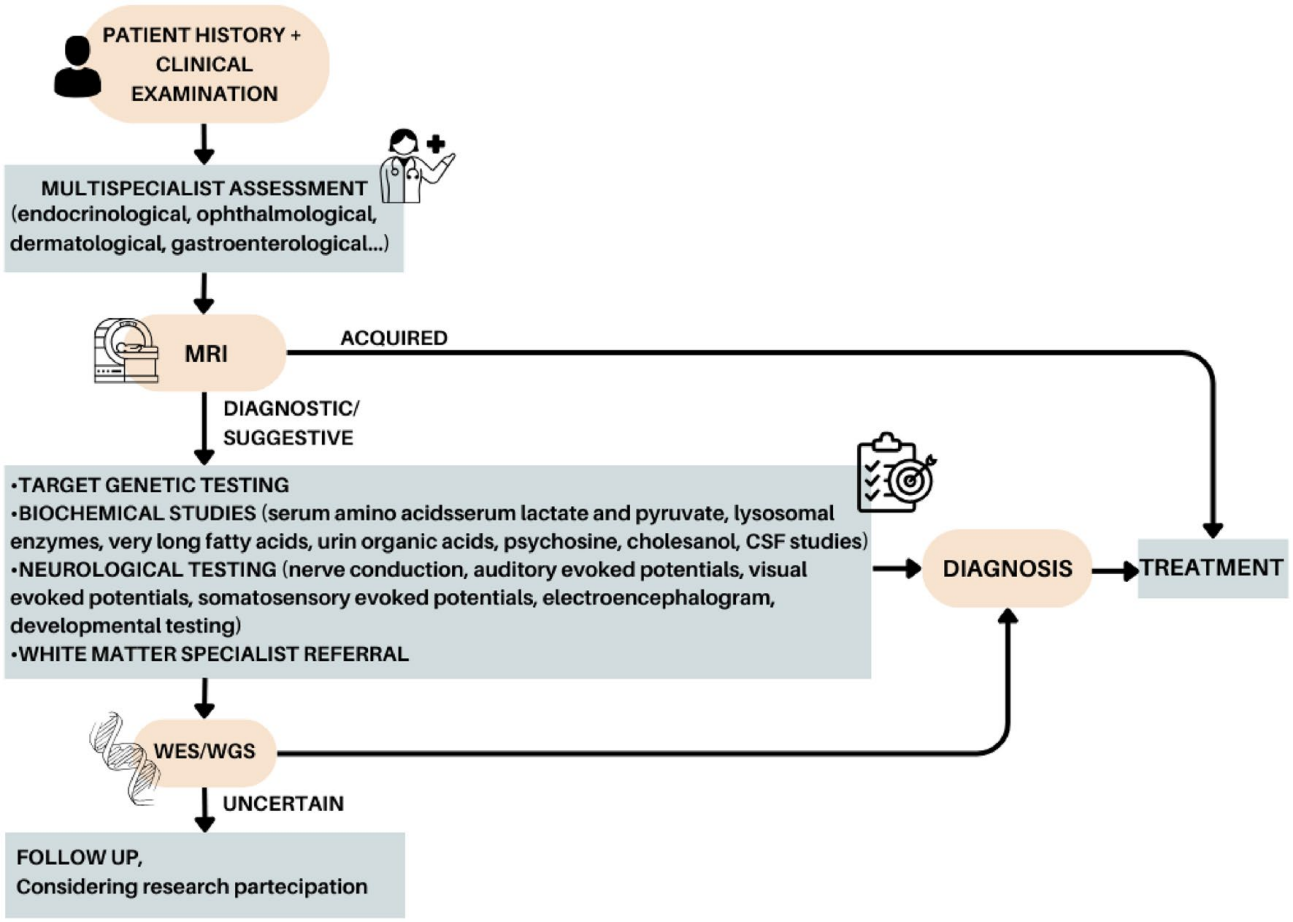
A functional approach for diagnosis of leukodystrophies

The diagnostic process for leukodystrophies begins with a thorough patient history and a comprehensive general and neurological examination. Because of the similarities in clinical findings among different types of leukodystrophies, it is crucial to combine these findings with specific brain MRI patterns. Additional diagnostic tools, including biochemical and metabolic tests, electrophysiological studies, ophthalmology consultation, neuroimaging, and genetic studies, can be used in parallel to enhance the accuracy of diagnosis in heritable white matter disorders. The integration of MRI, careful clinical evaluation, and next-generation genetic sequencing shows promise in reducing the number of unsolved cases (Fig. 3). Newborn screening can also play a pivotal role in early diagnosis and treatment [41].

*MRI* plays a central role in the diagnostic algorithms for leukodystrophies. Detailed analysis of MRI patterns can aid in identifying specific types of leukodystrophies.

Hypomyelinating leukodystrophies exhibit mild white matter abnormalities on T2-weighted imaging with iso- or hyperintense T1 signals, while demyelinating forms show prominent T2-weighted abnormalities and significant T1 hypointensity (Fig. 1). Adequate brain imaging protocols should be followed, including axial and sagittal T1-weighted, axial T2-weighted, and FLAIR sequences. Post-contrast T1-weighted images can provide additional information for certain conditions. Multiple MRI scans at intervals of at least 6 months may be necessary to better evaluate the progression and differential diagnosis.

While specific MRI findings can vary depending on the type and stage of the leukodystrophy, some typical findings may be observed. Cystic changes in the anterior temporal lobe are associated with several leukodystrophies such as AGS [39] and megalencephalic leukoencephalopathy with subcortical cysts (MLC) [33]. The corpus callosum is typically generally affected in MLD, Krabbe disease [35], and X-ALD [40], while the involvement of the inner rim of the anterior corpus callosum is suggestive of VWM [32]. Cystic degeneration is also observed in VWM [32] and AxD [31]. Prominent hypomyelination of the brainstem and the corticospinal tract is indicative of PMD [37]. Regions of demyelination alternating with relatively preserved myelinated areas give rise to a “tigroid pattern”, which is indicative of MLD [42].

While MRI is a sensitive technique for detecting brain white matter abnormalities, it has limitations and may not always provide conclusive results.

*Genetics testing* when considering the approach to genetic testing for leukodystrophies, several factors come into play. If a specific diagnosis is strongly suspected based on clinical symptoms and imaging findings, targeted genes or appropriate gene panels may be an initial step; however, if the diagnosis is uncertain or there is a suspicion of a broader genetic aetiology, WES or WGS becomes the test of choice [43]. WES has significantly improved the diagnostic yield of leukodystrophies, increasing the percentage of resolved cases by around 10% in recent years [4, 44]. Compared with the traditional step-by-step diagnostic approach, WES is more cost effective and delivers results within weeks or months, and should be commonly considered in clinical practice. It is important to note that WES may not detect all genetic variants, and an additional challenge associated with WES is the interpretation of variants of uncertain significance (VUS). VUS are genetic variants that lack sufficient evidence to determine their pathogenicity or benign nature. Further functional studies, segregation analysis within families, or the accumulation of additional evidence over time may be necessary to classify VUS as either pathogenic or benign. Negative findings on WES may indicate that the patient does not have a genetic disease, but clinical and MRI recognition of the disease along with targeted genetic testing may still be necessary. While WES-based gene-panel analysis is faster than open WES, it shares similar limitations. Genetic testing, including WES and WGS, should not be viewed as a standalone diagnostic tool.

Timely and accurate diagnosis of leukodystrophies is crucial for appropriate treatment options and participation in clinical trials.

*Metabolic testing and biomarkers* Biochemical and metabolic tests can provide guidance on the most appropriate genetic testing approach and help identify treatable entities, improving the accuracy of the final diagnosis in numerous leukodystrophies [5, 45, 46]. Recommended biochemical tests include very long-chain fatty acid (VLCFA) profiles, which are elevated in the plasma and tissues of patients with X-ALD [40, 47–50], and measurements of leukocyte lysosomal enzyme activity are used to investigate deficiencies in galactocerebrosidase (GALC) (Krabbe disease) [51] and arylsulfatase A (ARSA) activity (MLD) [52]. In some cases, enzymatic studies must be supported by biochemical assessments of substrate accumulation, for instance, elevated urine sulfatide levels are used as biomarkers to provide additional evidence for the diagnosis of MLD [53–55]. Furthermore, CTX is characterized biochemically by elevated levels in plasma cholestanol concentration (five- to ten-fold greater than normal), urine bile alcohol concentration, as well as plasma bile alcohol concentration. Disease-specific metabolic profiles can be used as biomarkers to help with early and definitive diagnosis, better disease prognostication, elucidating disease mechanisms, as well as monitoring therapeutic outcomes from specific treatments, especially with advancing therapeutic options [56–60]. For instance, a correlation between residual ARSA enzyme activity and the severity of clinical presentation was reported in MLD patients [61], and longitudinal assessments showed that there were decreases in dried blood spot (DBS) concentrations of psychosine, a substrate of the GALC enzyme, associated with natural disease progression and treatment with HSCT in Krabbe disease patients [62, 63]. Moreover, non-disease-specific and non-invasive biomarkers of neuroaxonal and astroglial injury, such as neurofilament light chain (NfL) and glial fibrillary acidic protein (GFAP) levels, can be widely applicable across leukodystrophies. Blood NfL levels were correlated with abnormalities on brain MRI in MLD [64], GFAP levels in the CSF of AxD patients were consistently elevated [65], and the potential of plasma NfL as a biomarker of spinal cord degeneration in adrenoleukodystrophy has been illustrated [66]. Additionally, mathematical models can be implemented to identify novel biomarkers for leukodystrophies, where an in silico systems biology-based modelling approach was used to identify a panel of 16 potential biomarker candidates for future validation in clinical cohorts to aid in prediction of early damage in MLD [67].

### Treatment

Currently, there are no disease-modifying treatments available for the majority of leukodystrophies. The treatment is mainly symptomatic and often palliative. Symptomatic treatment typically involves a multidisciplinary team of specialists across paediatrics, neurology, rehabilitation, orthopaedics, physical therapy, speech-language therapy, occupational therapy, social work, nutrition, pulmonary, sleep medicine, ophthalmology, audiology, and palliative care. Managing spasticity, a prevalent problem in leukodystrophies, can be accomplished through oral medications, such as baclofen, tizanidine, or diazepam, plus botulinum toxin. Anti-seizure medications can be used for treating seizures. It is important to pay close attention to possible respiratory complications and to diet and feeding methods to ensure sufficient caloric intake and to prevent aspiration. Furthermore, patients with leukodystrophies often require specific interventions (Table 3).

**Table 3:** Specific treatments in leukodystrophies

Leukodystrophies	Specific treatment	Gene therapy
X-ALD [68]	Hydrocortisone replacement (Addison syndrome)	HSCT (pre-symptomatic)
CTX	Chenodeoxycholic acid, inhibitors of HMG-CoA reductase	HSCT
Krabbe disease		HSCT
MLD	Cholecystectomy	HSCT arsa-cel
Canavan		AAV
4H syndrome	Growth hormone substitution	

*CTX* cerebrotendinous xanthomatosis, *MLD* metachromatic leukodystrophy, *X-ALD* X-linked adrenoleukodystrophy

*Gene therapy* is a therapeutic approach that involves introducing a functional copy of a gene into cells to achieve therapeutic effects [69]. Gene transfer can be performed ex vivo or in vivo. In ex vivo gene therapy, cells are taken from the patient's body and modified outside the body (in vitro), while in in vivo gene therapy, the gene transfer is performed directly inside the patient's body. Both approaches can be undertaken using various techniques, such as viral vectors (e.g. lentiviruses or adenoviruses) or non-viral methods (e.g. plasmid DNA or mRNA). Directly targeting the brain with gene therapy is becoming a viable option [70].

Bone marrow transplants or hematopoietic stem cell transplantation (HSCT) has shown successful outcomes in attenuating disease progression in leukodystrophies, characterized by the presence of cytotoxic metabolites in the brain (X-ALD, CTX, Krabbe) (Table 3). The rationale behind HSCT in these diseases involves the ability of hematopoietic cells to infiltrate the CNS and express the missing gene or enzyme necessary for the degradation of cytotoxic metabolites.

Lentiviruses have been used as a treatment in patients with pre-symptomatic MLD [71] with clinically relevant benefits [38, 72].

Gene therapy using adeno-associated viruses (AAVs) [73, 74] has several advantages over lentiviruses, including a broader tropism for CNS cell populations and the ability to cross the blood–brain barrier (for specific serotypes). An AAV1-GALC gene therapy study demonstrated reduced psychosine levels in the brain of a mouse model, indicating that delivery of the viral vector via the cerebroventricular system can decrease the rate of the disease progression in Krabbe disease [75]. Another study using a viral vector carrying the ASPA gene in patients with Canavan disease demonstrated safety and resulted in some clinical benefits [76]. However, it is possible that gene delivery alone may not be sufficient and may need to be combined with immunomodulation or silencing of the mutant gene.

Antisense oligonucleotides (ASOs) are RNA-based therapies that can alter protein expression (Canavan disease and PMD) [77].

CRISPR/Cas9 technology has not been studied on leukodystrophies, but it is a promising method for precise gene editing through homology-dependent repair mechanisms and can be used to repair disease-causing alleles by changing the DNA at the desired location of the chromosome.

#### Ongoing clinical trials

##### ION373 in Alexander disease (AxD) [78]

AxD is a progressive inherited disorder caused by mutations in the glial fibrillary acidic protein (*GFAP*) gene, resulting in astrocyte dysfunction. ION373 is a second-generation ASO therapy designed to target and prevent the production of GFAP. Although ION373 has not yet been evaluated in clinical trials, preclinical studies using ASOs targeting *GFAP* in rodent models of AxD have shown positive results, demonstrating phenotypic benefits. Additionally, further animal studies have confirmed and elucidated the safety and pharmacokinetic profiles of ION373.

The ION373-CS1 trial aims to evaluate the efficacy of ION373 in improving or stabilizing gross motor function in patients with AxD. The primary endpoint is the assessment of gross motor function, while the secondary endpoint focuses on the impact of ION373 on other symptoms of the disease.

##### VK0214 in X-linked adrenoleukodystrophy (X-ALD) [79]

X-ALD is an often-fatal metabolic disorder characterized by demyelination of brain and nerve cells. It is caused by mutations in the *ABCD1* gene, resulting in the dysfunction of the adrenoleukodystrophy protein (ALDP) and the accumulation of very long-chain fatty acids (VLCFAs). VK0214 (formerly MB-10866) is a thyroid beta receptor (TRβ) agonist that aims to normalize VLCFA metabolism, potentially impacting all forms of X-ALD. VK0214 has shown promising results in preclinical studies and has demonstrated safety, tolerability, and significant reductions in key lipids in healthy subjects in Phase 1 studies. The ongoing Phase 1b study of VK0214 involves male subjects with AMN. The study aims to evaluate the safety, tolerability, pharmacokinetics, pharmacodynamics, and efficacy of VK0214. The secondary and exploratory objectives include evaluating the impact of VK0214 on VLCFA levels in patients with AMN.

##### MIN-102 treatment for cerebral X-linked adrenoleukodystrophy [80–82]

cALD is the most severe form of X-ALD and is often fatal. MIN-102 (Leriglitazone) is a novel selective agonist of peroxisome proliferator-activated receptor gamma (PPARγ). It acts by binding to and activating the PPARγ receptors, which are nuclear receptors involved in regulating gene expression, that play a role in various biological processes, including glucose metabolism, lipid homeostasis, and inflammation. Activation of PPARγ by Leriglitazone, if administered prior to HSCT, can potentially have beneficial effects on the neuroinflammatory and neurodegenerative processes associated with the adrenoleukodystrophy disease pathway.

Notably, in the ongoing trial, the occurrence of new inflammatory lesions or growth of non-inflammatory lesions was observed exclusively in the placebo group, suggesting that MIN-102 treatment may have the potential in slowing the progression of cALD.

##### OTL-200 gene therapy for late juvenile metachromatic leukodystrophy (MLD) [83]

MLD is a rapidly progressing genetic disease characterized by loss of mobility, cognitive function, and early death. OTL-200 gene therapy (Libmeldy) shows the potential of improving mobility and cognitive function while addressing the enzyme deficiency caused by the disease. OTL-200 involves the use of ex vivo autologous CD34 + hematopoietic stem and progenitor cells collected from the patient's bone marrow or peripheral blood. These cells are then modified using a lentiviral vector, which inserts copies of the human ARSA complementary DNA into the cell genome. Once successfully engrafted, the genetically modified cells secrete functional ARSA enzyme, which helps break down or prevent the buildup of harmful substances in the brain. The effects of OTL-200 are expected to be long lasting.

##### rAAV-Olig001-ASPA gene therapy for Canavan disease (CAN-GT) [84]

CD is a fatal childhood genetic brain disease caused by mutations in the Aspartoacylase gene (*ASPA*), which disrupts the normal expression of Aspartoacylase (ASPA), a critical enzyme produced in oligodendrocytes. This enzyme is responsible for metabolizing the neurochemical *N*-acetylaspartate (NAA). In CD, the impaired metabolism of NAA by oligodendrocytes leads to its accumulation in the brain, resulting in detrimental effects on bioenergetics, myelin production, and overall brain health. CD symptoms typically become apparent several months after birth, characterized by poor head control, abnormally large head size, impaired eye tracking, excessive irritability, diminished muscle tone, and delays in motor milestones such as rolling, sitting, and walking. As the disease progresses, seizures, spasticity, swallowing difficulties, and motor deterioration worsen, often leading to life-threatening complications by around 10 years of age. rAAV-Olig001-ASPA gene therapy aims to restore ASPA function in the oligodendrocytes of Canavan patients' brains, thereby rescuing NAA metabolism in its natural cellular compartment and supporting myelination and remyelination processes carried out by resident oligodendrocytes. This therapeutic approach specifically targets oligodendrocytes; it is administered through a neurosurgical procedure involving the direct delivery of gene therapy to the affected regions of the brain. The ongoing clinical trial assessing this therapy utilizes longitudinal clinical assessments and brain imaging as outcome measures. Promising results from a Myrtelle clinical study demonstrated significant improvements in myelin, white matter, grey matter, and total brain volume, as well as reductions in cerebrospinal fluid volume, compared to untreated CD patients. No serious drug-related adverse events have been reported to date.

## Conclusion

The field of leukodystrophies has made significant advances of late including improved diagnostic approaches, more accurate prognostic predictions, and a myriad of novel therapeutic options. A better characterization of the underlying pathomechanisms along with the identification of appropriate biomarkers is crucial for the design of effective targeted treatments. It is essential for healthcare professionals and researchers to stay up to date on these advancements and incorporate them into clinical practice to enhance the diagnosis, management, and treatment of patients with leukodystrophies. This review offers healthcare professionals an understanding of leukodystrophies, including clinical and genetic aspects. It also highlights ongoing clinical trials and emerging therapies with the potential to improve patient management and outcomes. Continued research and collaboration are vital for further progress in understanding and addressing the challenges associated with these complex diseases.

### Supplementary Information

Below is the link to the electronic supplementary material.

Supplementary file1 (DOCX 76 KB)

## Data Availability

The authors declare that the data supporting the findings of this study are available within the paper and its Supplemental Files.

## Abbreviations

18q-: 18Q deletion syndrome
4H: 4H syndrome
AMN: Adrenomyeloneuropathy
ALSP: Adult-onset leukoencephalopathy with axonal spheroids and pigmented glia
AGS: Aicardi–Goutieres syndrome
AxD: Alexander disease
ADLD: Autosomal dominant leukodystrophy with autonomic disease
CADASIL: Cerebral autosomal dominant arteriopathy with subcortical infarcts and leukoencephalopathy
CRMCC: Cerebroretinal microangiopathy with calcifications and cysts
CTX: Cerebrotendinous xanthomatosis
CACH: Childhood ataxia with central nervous system hypomyelination
HDLS: Hereditary diffuse leukoencephalopathy with spheroids
HLD1: Hypomyelinating leukodystrophy-1
HLD10: Hypomyelinating leukodystrophy-10
HLD11: Hypomyelinating leukodystrophy-11
HLD12: Hypomyelinating leukodystrophy-12
HLD13: Hypomyelinating leukodystrophy-13
HLD14: Hypomyelinating leukodystrophy-14
HLD15: Hypomyelinating leukodystrophy-15
HLD16: Hypomyelinating leukodystrophy-16
HLD17: Hypomyelinating leukodystrophy-17
HLD18: Hypomyelinating leukodystrophy-18
HLD2: Hypomyelinating leukodystrophy-2
HLD20: Hypomyelinating leukodystrophy-20
HLD21: Hypomyelinating leukodystrophy-21
HLD22: Hypomyelinating leukodystrophy-22
HLD23: Hypomyelinating leukodystrophy-23 with ataxia, deafness, liver dysfunction, and dilated cardiomyopathy
HLD24: Hypomyelinating leukodystrophy-24
HLD25: Hypomyelinating leukodystrophy-25
HLD26: Hypomyelinating leukodystrophy-26 with chondrodysplasia
HLD3: Hypomyelinating leukodystrophy-3
HLD4: Hypomyelinating leukodystrophy-4/mitochondrial Hsp60 chaperonopathy
HLD6/HABC: Hypomyelinating leukodystrophy-6
HLD7: Hypomyelinating leukodystrophy-7 with or without oligodontia and/or hypogonadotropic hypogonadism
HLD9: Hypomyelinating leukodystrophy-9
LTBL: Hypomyelination with brainstem and leukoencephalopathy with thalamus and brainstem involvement and high lactate
HBSL: Hypomyelination with brainstem and spinal cord involvement and leg spasticity
HCC: Hypomyelination with congenital cataract
HLD5: Hypomyelinating leukodystrophy-5
LCC: Leukodystrophy with calcifications and cyst
LBSL: Leukoencephalopathy with brainstem and spinal cord involvement and lactate elevation
MLC: Megalencephalic leukoencephalopathy with subcortical cysts
MLD: Metachromatic leukodystrophy
MSD: Multiple sulfatase deficiency
ODDD: Oculodentodigital dysplasia
PMD: Pelizaeus–Merzbacher disease
PMLD1: Pelizaeus–Merzbacher-like disease
POLD: Pigmentary orthochromatic leukodystrophy
POLR3: POLR3-related disorders
SLS: Sjögren–Larsson syndrome
SPAX8: Spastic ataxia 8 with hypomyelinating leukodystrophy
VWM: Vanishing white matter disease
RVCL-S: Retinal vasculopathy with cerebral leukodystrophy
X-ALD: X-linked adrenoleukodystrophy
