# Supplementary material for: Update on leukodystrophies and developing trials
Source: J Neurol. 2023 Sep 27;271(1):593–605. doi: 10.1007/s00415-023-11996-5 (PMC10770198; doi:10.1007/s00415-023-11996-5)
Supplement: Supplementary file 1 — Supplementary file1 (DOCX 76 KB) [file 415_2023_11996_MOESM1_ESM.docx]

**Supplementary Material**

**Table 1** Classifications of leukodystrophies based on inheritance patterns, with additional information such as the description of genes involved, clinical manifestations, MRI findings, and pathophysiology.

| LEUKODYSTROPHIES | GENE | INHERITANCE | PHATOPHISIOLOGY | AGE OF ONSET | MAIN CLINICAL FEATURES | TREATMENT OPTIONS | MRI | OTHER INVESTIGATIONS |
| --- | --- | --- | --- | --- | --- | --- | --- | --- |
| Adult-onset leukoencephalopathy with axonal spheroids and pigmented glia (ALSP) - hereditary diffuse leukoencephalopathy with spheroids (HDLS) -pigmentary orthochromatic leukodystrophy (POLD) | *CSF1R* | AD | Unknown | 50-60 yo | Rapidly progressive neurodegenerative disorder characterized by variable behavioral, cognitive, motor changes. | Symptomatic treatments, Physical, occupational and speech therapy^1^  TRIAL: VGL101 | Frontal predominance. May be patchy and asymmetric. May extend to the PLIC or pyramidal tracts later in the disease. |  |
| Hypomyelination with atrophy of the basal ganglia and cerebellum (H-ABC) / Hypomyelinating leukodystrophy-6 (HLD6) | *TUBB4A* | AD |  | Infancy or early childhood | Delayed motor development and gait instability, followed by extrapyramidal movement disorders | Symptomatic treatments, supplementation with Calcium and vitamin D^2^ |  |  |
| Hypomyelinating leukodystrophy-16 (HLD16) | *TMEM106B* | AD |  | Infancy | Hypotonia, nystagmus, and mildly delayed motor development | Symptomatic treatments^3^ |  |  |
| Hypomyelinating leukodystrophy-22 (HLD22) | *CLDN11* | AD |  | Childhood | Global developmental delay, mild intellectual impairment, marked motor impairment, dysarthria | Symptomatic treatments^4^ |  |  |
| Hypomyelinating leukodystrophy-24 (HLD24) | *ATP11A* | AD |  | Early infancy | Global developmental delay and neurologic deterioration | Symptomatic treatments^5^ |  |  |
| Hypomyelinating leukodystrophy-25 (HLD25) | *TMEM163* | AD |  | Neonatal, infancy | Horizontal nystagmus, hypotonia, and global developmental delay | Symptomatic treatment^6^ |  |  |
| Alexander disease (AxD) | *GFAP* | AD | Gain-of-function variants in GFAP → GFAP accumulation | Neonatal, infantile, juvenile, and adult | Seizures, megalencephaly, developmental delay, and spasticity or bulbar or pseudobulbar symptoms, spasticity, feeding difficulties | Symptomatic treatments^7^  TRIAL: ION373 | In the infantile form, there is T2/FLAIR hyperintensity primarily observed in the frontal regions, basal ganglia, thalamus, and brainstem. Periventricular regions show T2 hypointensity. Contrast enhancement is seen in the periventricular regions, brainstem, and ventricular garlands.  In the adult-onset form, there is involvement of the medulla and cervical spine, characterized by increased T2 signal and atrophy. Progressive atrophy of the medulla and cervical spinal cord results in the "tadpole sign." Rare lesions may also be present in the pontine, middle cerebellar peduncle, inferior cerebellar peduncle, superior cerebellar peduncle, and supratentorial regions. Space-occupying lesions may also be observed. | Presence of Rosenthal fibers in Astrocytic processes, and there is an increase in GFAP levels in the CSF. |
| Autosomal Dominant Leukodystrophy with Autonomic disease (ADLD) | *LMNB1* | AD |  | Adult (40-50 yo) | Autonomic abnormalities, pyramidal and cerebellar dysfunction, symmetric demyelination of the CNS | Symptomatic treatments^8^ |  |  |
| 18q deletion syndrome |  | AD |  | Infancy/early childhood | Variable, impaired intellectual development, short stature, hypotonia, hearing impairment, foot deformities. | Symptomatic treatments^9^ |  |  |
| Metachromatic leukodystrophy (MLD) | *ARSA* | AR | Insufficient enzymatic activity of **arylsulfatase A** leads to the accumulation of sulfatides in the peripheral and central nervous systems, as well as kidneys and the gallbladder. | Late infantile – early childhood (<30 months), juvenile (30 months-puberty), and adult forms (>17 yo) | Late infantile: motor symptoms, regression motor and speech, weakness, hypotonia, convulsions. Late juvenile: regression, mental status changes, behavioral disturbances, personality change. Adult-onset: cognitive/behavioral deficits, psychiatric manifestations, spasticity, seizures, movement disorders, cerebellar ataxia, demyelinating neuropathy, gallbladder polyps / biliary colic | Symptomatic treatments. HSCT / Bone marrow transplantation  before onset of neurological symptoms^10,11^  TRIAL: OTL-200 | •Diffuse WM abnormalities with an appearance of radiating stripes (tigroid  pattern)  •Involvement of the CC and pyramidal tracts  •Sparing of subcortical WM  Adult  • Frontal + pyramidal tract predominance | Leukocytes exhibit reduced arylsulfatase A activity, while the presence of sulfatides in urine is elevated. |
| Pol-III related disorders (4H syndrome, Hypomyelinating leukodystrophy-7, with or without oligodontia and/or hypogonadotropic hypogonadism; HLD7) | *POLR3A*  *POLR3B* | AR |  | Early infancy, childhood | Dystonia, nystagmus, ataxia, spasticity, mild cognitive deficit, hypodontia, delayed or absent puberty, hypomyelination, hypogonadotropic hypogonadism, hypodontia, dental and endocrine abnormalities | Symptomatic treatment^12^ |  |  |
| Hypomyelinating leukodystrophy-21 (HLD21) | *POLR3K* | AR |  | Infancy | global developmental delay, loss of motor, speech, and cognitive milestones | Symptomatic treatments^13^ |  |  |
| Hypomyelinating leukodystrophy-11 (HLD11) | *POLR1C* | AR |  | Neonatal, early infancy | Psychomotor developmental delay, dental abnormalities, possibly hypogonadotropic hypogonadism | Symptomatic treatment^14^ |  |  |
| Globoid cell leukodystrophy/ Krabbe disease | *GALC* | AR | Deficiency of galactocerebrosidase enzyme results in the lysosomal accumulation of galactocerebroside and psychosine. These accumulated substances are toxic to oligodendrocytes, leading to their impaired function and subsequent pathological effects. | Infantile, juvenile, adult onset (<20 yo) | Early Infantile: extreme irritability, failure to thrive, developmental arrest or regression, stiffness, seizures, absence of voluntary movement, severe motor, mental deterioration. Late-onset infantile: psychomotor regression, ataxia, slow progression. Juvenile onset: visual impairment, ataxia, slow progression. Adult onset: spastic paraparesis | Symptomatic treatments. HSCT / Bone marrow transplantation  before onset of neurological symptoms^15^ | CT:  hyperdensity observed in the basal ganglia and thalamus.  MRI:  In MRI imaging, the predominant involvement is seen in the parietooccipital region or corticospinal tract. There is early involvement of the posterior fossa, particularly the dentate nucleus. Additionally, there is thickening of the optic nerves and chiasm. | Leukocytes exhibit reduced galactocerebrosidase activity, while there is an increase in galactocerebrosidase levels in CSF. |
| eIF2B related disorder (Vanishing White Matter Disease (VWM) or Childhood ataxia with central nervous system hypomyelination (CACH)) | *EIF2B1, EIF2B2, EIF2B3, EIF2B4, EIF2B5* | AR | eIF2B is a rate-limiting factor for protein translation that is modulated by the endoplasmic reticulum stress response. | Antenatal, infantile, childhood, juvenile | Early childhood presentation with deteriorations after falls or acute illness (AMS, acute paresis, or hypotonia). Progressive course with partial recovery between episodes  paucisymptomatic adult forms (spastic paraparesis, cognitive decline, ovarian failure)  • Childhood-onset progressive ataxia, Seizures, cognitive deficit, spasticity, optic atrophy  Episodic deterioration with stress or fever, +/- episodes of coma  • Premature ovarian failure  Progressive cerebellar ataxia, spasticity, cognitive impairment | Symptomatic treatments^16^ | Diffuse T2 hyperintensity, followed by rarefaction, cystic degeneration, and replacement by fluid resembling CSF. Pattern of radiating tissue strands can be observed, on sagittal T1 imaging, indicating the preservation of tissue in certain areas. |  |
| Megalencephalic leukoencephalopathy with subcortical cysts (MLC) | *MLC1, HEPACAM* | AR |  | Early childhood | Megalencephaly, mild motor developmental delay and seizures. Gradual onset of ataxia, spasticity possible extrapyramidal findings | Symptomatic treatment^17^ |  |  |
| Canavan disease | *ASPA* | AR |  | Early infancy | Lethargy, hypotonia, megalencephaly, severe neurological deterioration, spasticity, blindness, seizures, exaggerated startle response, death | Symptomatic treatments  gene therapy using lentiviruses and recombinant adeno-associated viruses^18^  TRIAL: rAAV-Olig001-ASPA |  |  |
| Cerebrotendinous xanthomatosis (CTX)^19^ | *CYP27A1* | AR |  | Puberty, adulthood | Progressive neurologic dysfunction, premature atherosclerosis, cataracts, optic disk paleness, diarrhea, Premature atherosclerosis, Osteoporosis, bone fractures | Chenodeoxycholid acid, inhibitors of HMG-CoA reductase |  |  |
| Hypomyelination with Brainstem and Spinal Cord involvement and Leg Spasticity (HBSL) | *DARS1* | AR |  | Infancy early childhood | Delayed motor development and nystagmus; some may have mild mental retardation | Symptomatic treatment^20^ |  |  |
| Leukoencephalopathy with brainstem and spinal cord involvement and lactate elevation (LBSL)^21^ | *DARS2* | AR |  | Infantile (rare), juvenile onset, adult-onset | Slowly progressive cerebellar ataxia, spasticity, and dorsal column dysfunction, sometimes with a mild cognitive deficit or decline | Symptomatic treatments |  |  |
| Hypomyelinating leukodystrophy-2 (HLD2)/ Pelizaeus-Merzbacher-Like Disease (PMLD1)^22^ | *GJC2/*  *GJA12* | AR |  | Early infancy | nystagmus, impaired motor development, ataxia, choreoathetotic movements, dysarthria, and progressive spasticity | Symptomatic treatments |  |  |
| Spastic ataxia 8, autosomal recessive, with hypomyelinating leukodystrophy (SPAX8)^23,24^ | *NKX6-2* | AR |  | Neonatal, childhood | Hypotonia, ataxia, spasticity, pyramidal syndrome, dystonia, dysarthria, abnormal eye movements | Symptomatic treatments |  |  |
| Hypomyelinating leukodystrophy-3 (HLD3)^25^ | *AIMP1* | AR |  | Early infancy | Global developmental delay, lack of development, lack of speech acquisition, peripheral spasticity | Symptomatic treatments |  |  |
| Mitochondrial Hsp60 chaperonopathy/ Hypomyelinating leukodystrophy-4 (HLD4)^26^ | *HSPD1* | AR |  | Neonatal, early infancy | Hypotonia, nystagmus, psychomotor developmental delay, prominent spasticity, developmental arrest or regression, seizures, malnutrition, and growth failure. | Symptomatic treatments |  |  |
| Hypomyelination with congenital cataract (HCC) / Hypomyelinating leukodystrophy-5 (HLD5)^27,28^ | *HYCC1* | AR |  | Neonatal | Congenital bilateral cataract, progressive pyramidal, cerebellar impairment | Symptomatic treatments |  |  |
| Hypomyelinating leukodystrophy-9 (HLD9) | *RARS1* | AR |  | Infancy or early childhood | Psychomotor developmental delay, spasticity, nystagmus, ataxia | Symptomatic treatments |  |  |
| Hypomyelinating leukodystrophy-10 (HLD10) | *PYCR2* | AR |  | Neonatal, early infancy | Progressive microcephaly, sever psychomotor developmental delay | Symptomatic treatments |  |  |
| Hypomyelinating leukodystrophy-12 (HLD12) | *VPS11* | AR |  | Early infancy | Severe psychomotor developmental delay or absence, microcephaly, spasticity | Symptomatic treatments  p70S6K phosphatases inhibitors (PP1C and PP2A).  Okadaic acid-like compounds.^29^ |  |  |
| Hypomyelinating leukodystrophy-13 (HLD13) | *HIKESHI* | AR |  | Infancy | Psychomotor developmental delay, axial hypotonia, and spasticity | Symptomatic treatments.  Ketogenic diet and iron-rich food ^30^ |  |  |
| Hypomyelinating leukodystrophy-14 (HLD14) | *UFM1* | AR |  | Neonatal, early infancy | Hypotonia, almost absence of motor, cognitive and language development, spasticity, intractable seizures. Possible hearing loss and/or blindness, feeding or ventilatory support | Symptomatic treatments  Ketogenic diet for drug- resistant seizures^31^ |  |  |
| Hypomyelinating leukodystrophy-15 (HLD15) | *EPRS* | AR |  | 10-20 yo | Dystonia, ataxia, spasticity, dysphagia, frequent optic atrophy, and hearing loss | Symptomatic treatments^32^ |  |  |
| Hypomyelinating leukodystrophy-17 (HLD17) | *AIMP2* | AR |  | Early infancy | No milestone, early-onset multifocal seizures, spasticity, poor overall growth, severe microcephaly, death | Symptomatic treatments^,33,34^ |  |  |
| Hypomyelinating leukodystrophy-18 (HLD18) | *DEGS1* | AR |  | Early infancy | Poor psychomotor development, including inability to sit or walk independently in the more severe cases, as well as poor or absent speech, dystonia, and spasticity | Symptomatic treatments Fingolimod (FTY720) treatment shows promise in a zebrafish model^35^ |  |  |
| Hypomyelinating leukodystrophy-20 (HLD20) | *CNP* | AR |  | 12- 16 months old | Loss of developmental milestones after normal early development | Symptomatic treatments ^36^ |  |  |
| Hypomyelinating leukodystrophy-23 with ataxia, deafness, liver dysfunction, and dilated cardiomyopathy (HLD23) | *RNF220* | AR |  | Early childhood | Motor development and ataxic gait, spastic paraplegia with loss of ambulation in the first decades of life. Additional features include progressive sensorineural hearing loss resulting in deafness, hepatic dysfunction with elevated liver enzymes, and dilated cardiomyopathy that results in death in the first or second decades. | Symptomatic treatment^37^ |  |  |
| Hypomyelinating leukodystrophy-26 with chondrodysplasia (HLD26) | *SLC35B2* | AR |  | Neonatal, infancy, childhood | Severe psychomotor delay, involving motor and expressive language development, with cerebral and cerebellar atrophy and corpus callosum hypoplasia. In addition, patients show pre- and postnatal growth retardation, early-onset scoliosis, and dislocations of large joints | Symptomatic treatments^38^ |  |  |
| Sjögren-Larsson syndrome (SLS) | *ALDH3A2* | AR |  | <24 months | Ichthyosis, intellectual disability, spastic paraparesis, macular dystrophy, retinal glistening white dots, seizures, leukoencephalopathy | Symptomatic treatments, daily skin care with emollients and retinoids^39^  Gene therapy: utilizing an adenovirus vector to induce in vitro FALDH activity in keratinocytes has shown promising results.^40^ |  |  |
| Aicardi-Goutieres syndrome (AGS) | *TREX1, ADAR, IFIH1, RNASEH2A, RNASEH2B, RNASEH2C, SAMHD1, LSM11, RNU7-1* | AR (mostly) / AD / Sporadic | Autoinflammatory leukodystrophy caused by variants in genes associated with DNA and RNA processing. | Early infancy, infancy | Progressive microcephaly, severe encephalopathy, developmental delay, spasticity, axial hypotonia, marked startle, dystonic posturing, profound psychomotor retardation, and often death in early childhood. Sterile pyrexias, may mimic congenital infection, especially CMV (both clinical picture and imaging). Hypothyroidism, hepatitis, glaucoma, thrombocytopenia, chilblains - small purple-red or white blistering lesions on the toes (inflammatory skin lesions), fingers, helix, pressure areas. | Symptomatic treatments^41^  Different drugs have been explored:  Janus kinase inhibitors (baricitinib, ruxolitinib, tofacitinib) and reverse transcriptase inhibitors^42, 43, 44^ |  | Systemic inflammation |
| Oculodentodigital dysplasia (ODDD)^45^ | *GJA1* | AD/ AR |  | Childhood | typical facial appearance, variable involvement of the eyes (microcornea, cornea opacity and glaucoma) dentition, and fingers | Symptomatic treatments |  | Cardiac screening should be considered |
| Pelizaeus-Merzbacher disease (PMD)  Hypomyelinating leukodystrophy-1 (HLD1) | *PLP1* | XL |  | Infancy/ juvenile | Nystagmus in first weeks of life, dystonia, developmental delay progressing to spasticity, with ataxia, extrapyramidal features, cognitive impairment, peripheral neuropathy in subtype PLP1 null syndrome | Symptomatic treatments  Curcumin, an extract of turmeric with anti-oxidative effect for motor improvement^46^ | • Prototypic hypomyelinating disorder: increased T1 and T2 signals  • Severely reduced amount of white matter/myelin (e.g., thin CC)  • Some myelin can still be observed in specific areas such as the posterior limb of the internal capsule (PLIC), optic radiation, brainstem, and cerebellar hemispheres.  • “Tigroid” aspect- small, myelinated areas surrounding blood vessels |  |
| X-linked Adrenoleukodystrophy (X-ALD) | *ABCD1* | XL | The deficiency of the peroxisomal transporter ABCD1 causes the accumulation of VLCFA. These accumulated fatty acids, along with the disruption of the cerebralmicrovasculature, and the activation of the immune system, contribute to pathological changes in the brain, spinal cord, and adrenal glands. | Childhood, adolescent, adult cerebral | Adrenal insufficiency (Addison disease) Behavioural changes, changes in fine motor skills, cognitive dysfunction, gait changes, visual loss, rapid deterioration, spastic quadriparesis and dementia. | Symptomatic treatments  urgent hormone replacement; hematopoietic transplantation /bone marrow transplantation  TRIALS: VK0214, MIN-102 | T2 hyperintensities in the splenium of the corpus callosum with parietooccipital predominance and sparing the arcuate fibers. Contrast enhancement at the border of the inner and outer zones of the lesion | Serum VLCFA levels.  Newborn screening is based on the measurement of C26:0 lysophosphatidylcholine (26:0-lyso-PC) in dried blood spots.^47^ |

*Cerebrospinal fluid (CSF), glial fibrillary acidic protein (GFAP), Haematopoietic stem cell transplantation (HSCT) and very long-chain fatty acids (VLCFA).*

**Supplementary References**

1. Papapetropoulos S, Pontius A, Finger E, et al. Adult-Onset Leukoencephalopathy With Axonal Spheroids and Pigmented Glia: Review of Clinical Manifestations as Foundations for Therapeutic Development. *Front Neurol*. 2022;12:788168. doi:10.3389/fneur.2021.788168

2. Nahhas N, Conant A, Hamilton E, et al. TUBB4A-Related Leukodystrophy. In: Adam MP, Mirzaa GM, Pagon RA, et al., eds. *GeneReviews®*. University of Washington, Seattle; 1993. Accessed June 25, 2023. http://www.ncbi.nlm.nih.gov/books/NBK395611/

3. Alotaibi L, Alqasmi A. Identification of a de novo Mutation in TMEM106B in a Saudi Child Causes Hypomyelination Leukodystrophy. *Glob Med Genet*. 2023;10(01):038-041. doi:10.1055/s-0043-1764370

4. Riedhammer KM, Stockler S, Ploski R, et al. *De novo* stop-loss variants in *CLDN11* cause hypomyelinating leukodystrophy. *Brain*. 2021;144(2):411-419. doi:10.1093/brain/awaa410

5. Segawa K, Kikuchi A, Noji T, et al. A sublethal ATP11A mutation associated with neurological deterioration causes aberrant phosphatidylcholine flipping in plasma membranes. *J Clin Invest*. 2021;131(18):e148005. doi:10.1172/JCI148005

6. Yan H, Yang S, Hou Y, et al. Functional Study of TMEM163 Gene Variants Associated with Hypomyelination Leukodystrophy. *Cells*. 2022;11(8):1285. doi:10.3390/cells11081285

7. Srivastava S, Waldman A, Naidu S. Alexander Disease. In: Adam MP, Mirzaa GM, Pagon RA, et al., eds. *GeneReviews®*. University of Washington, Seattle; 2020. Accessed June 25, 2023. http://www.ncbi.nlm.nih.gov/books/NBK1172/

8. Raininko R, Gosky M, Padiath QS. LMNB1-Related Autosomal Dominant Leukodystrophy. In: Adam MP, Mirzaa GM, Pagon RA, et al., eds. *GeneReviews®*. University of Washington, Seattle; 2016. Accessed June 25, 2023. http://www.ncbi.nlm.nih.gov/books/NBK338165/

9. Hale DE, Cody JD, Baillargeon J, Schaub R, Danney MM, Leach RJ. The Spectrum of Growth Abnormalities in Children with 18q Deletions ^1^. *J Clin Endocrinol Metab*. 2000;85(12):4450-4454. doi:10.1210/jcem.85.12.7016

10. Yan H, Helman G, Murthy SE, et al. Heterozygous Variants in the Mechanosensitive Ion Channel TMEM63A Result in Transient Hypomyelination during Infancy. *Am J Hum Genet*. 2019;105(5):996-1004. doi:10.1016/j.ajhg.2019.09.011

11. Sessa M, Lorioli L, Fumagalli F, et al. Lentiviral haemopoietic stem-cell gene therapy in early-onset metachromatic leukodystrophy: an ad-hoc analysis of a non-randomised, open-label, phase 1/2 trial. *The Lancet*. 2016;388(10043):476-487. doi:10.1016/S0140-6736(16)30374-9

12. Bernard G, Vanderver A. POLR3-Related Leukodystrophy. In: Adam MP, Mirzaa GM, Pagon RA, et al., eds. *GeneReviews®*. University of Washington, Seattle; 2012. Accessed June 25, 2023. http://www.ncbi.nlm.nih.gov/books/NBK99167/

13. Dorboz I, Dumay-Odelot H, Boussaid K, et al. Mutation in *POLR3K* causes hypomyelinating leukodystrophy and abnormal ribosomal RNA regulation. *Neurol Genet*. 2018;4(6):e289. doi:10.1212/NXG.0000000000000289

14. Kashiki H, Li H, Miyamoto S, et al. *POLR1C* variants dysregulate splicing and cause hypomyelinating leukodystrophy. *Neurol Genet*. 2020;6(6):e524. doi:10.1212/NXG.0000000000000524

15. Wenger DA, Luzi P, Rafi MA. Advances in the Diagnosis and Treatment of Krabbe Disease. *Int J Neonatal Screen*. 2021;7(3):57. doi:10.3390/ijns7030057

16. Deginet E, Tilahun R, Bishaw S, Eshetu K, Moges A. Probable Vanishing White Matter Disease: A Case Report and Literature Review. *Ethiop J Health Sci*. 2021;31(6):1307-1310. doi:10.4314/ejhs.v31i6.28

17. Blattner R, Von Moers A, Leegwater PA, Hanefeld FA, Van Der Knaap MS, Köhler W. Clinical and Genetic Heterogeneity in Megalencephalic Leukoencephalopathy with Subcortical Cysts (MLC). *Neuropediatrics*. 2003;34(4):215-218. doi:10.1055/s-2003-42210

18. Wei H, Moffett JR, Amanat M, et al. The pathogenesis of, and pharmacological treatment for, Canavan disease. *Drug Discov Today*. 2022;27(9):2467-2483. doi:10.1016/j.drudis.2022.05.019

19. Nie S, Chen G, Cao X, Zhang Y. Cerebrotendinous xanthomatosis: a comprehensive review of pathogenesis, clinical manifestations, diagnosis, and management. *Orphanet J Rare Dis*. 2014;9(1):179. doi:10.1186/s13023-014-0179-4

20. Zhu J, Guo X, Ran N, et al. Leukoencephalopathy hypomyelination with brainstem and spinal cord involvement and leg spasticity caused by DARS1 mutations. *Front Genet*. 2023;13:1009230. doi:10.3389/fgene.2022.1009230

21. Ngo J, Prokop JW, Umfleet J, Seaver LH. Perinatal Manifestations of *DARS2* -Associated Leukoencephalopathy With Brainstem and Spinal Cord Involvement and Lactate Elevation (LBSL). *Child Neurol Open*. 2021;8:2329048X2110191. doi:10.1177/2329048X211019173

22. Komachali SR, Sheikholeslami M, Salehi M. A novel mutation in GJC2 associated with hypomyelinating leukodystrophy type 2 disorder. *Genomics Inform*. 2022;20(2):e24. doi:10.5808/gi.22008

23. Singh R, Samanta D. Pelizaeus-Merzbacher Disease. In: *StatPearls*. StatPearls Publishing; 2023. Accessed August 30, 2023. http://www.ncbi.nlm.nih.gov/books/NBK560522/

24. Chelban V, Kaya N, Alkuraya F, Houlden H. NKX6-2-Related Disorder. In: Adam MP, Everman DB, Mirzaa GM, et al., eds. *GeneReviews®*. University of Washington, Seattle; 1993. Accessed February 25, 2023. http://www.ncbi.nlm.nih.gov/books/NBK531509/

25. Takeuchi Y, Tanaka M, Okura N, et al. Rare Neurologic Disease-Associated Mutations of AIMP1 Are Related with Inhibitory Neuronal Differentiation Which Is Reversed by Ibuprofen. *Medicines*. 2020;7(5):25. doi:10.3390/medicines7050025

26. Cömert C, Brick L, Ang D, et al. A recurrent de novo *HSPD1* variant is associated with hypomyelinating leukodystrophy. *Mol Case Stud*. 2020;6(3):a004879. doi:10.1101/mcs.a004879

27. Wolf NI, Biancheri R, Zara F, et al. Hypomyelination and Congenital Cataract. In: Adam MP, Mirzaa GM, Pagon RA, et al., eds. *GeneReviews®*. University of Washington, Seattle; 1993. Accessed June 25, 2023. http://www.ncbi.nlm.nih.gov/books/NBK2587/

28. Kraoua I, Bouyacoub Y, Drissi C, et al. Hypomyelination and Congenital Cataract: Clinical, Imaging, and Genetic Findings in Three Tunisian Families and Literature Review. *Neuropediatrics*. 2021;52(04):302-309. doi:10.1055/s-0041-1728654

29. Matsumoto N, Miyamoto Y, Hattori K, et al. PP1C and PP2A are p70S6K Phosphatases Whose Inhibition Ameliorates HLD12-Associated Inhibition of Oligodendroglial Cell Morphological Differentiation. *Biomedicines*. 2020;8(4):89. doi:10.3390/biomedicines8040089

30. Singh S, Mishra A, Murthy C, et al. A Rare Case of Hypomyelinating Leukodystrophy and Its Management: A Case Report and Literature Review. *Cureus*. Published online March 21, 2023. doi:10.7759/cureus.36471

31. Ünalp A, Köse M, Karaoğlu P, Güzin Y, Yılmaz Ü. A rare case of hypomyelinating leukodystrophy-14 benefiting from ketogenic diet therapy. *Turk J Pediatr*. 2022;64(4):747. doi:10.24953/turkjped.2021.1662

32. Sawaguchi S, Goto M, Kato Y, et al. Hypomyelinating Leukodystrophy 15 (HLD15)-Associated Mutation of EPRS1 Leads to Its Polymeric Aggregation in Rab7-Positive Vesicle Structures, Inhibiting Oligodendroglial Cell Morphological Differentiation. *Polymers*. 2021;13(7):1074. doi:10.3390/polym13071074

33. McInerney-Leo AM, Duncan EL. Massively Parallel Sequencing for Rare Genetic Disorders: Potential and Pitfalls. *Front Endocrinol*. 2020;11:628946. doi:10.3389/fendo.2020.628946

34. Shukla A, Das Bhowmik A, Hebbar M, et al. Homozygosity for a nonsense variant in AIMP2 is associated with a progressive neurodevelopmental disorder with microcephaly, seizures, and spastic quadriparesis. *J Hum Genet*. 2018;63(1):19-25. doi:10.1038/s10038-017-0363-1

35. Pant DC, Dorboz I, Schluter A, et al. Loss of the sphingolipid desaturase DEGS1 causes hypomyelinating leukodystrophy. *J Clin Invest*. 2019;129(3):1240-1256. doi:10.1172/JCI123959

36. Al-Abdi L, Al Murshedi F, Elmanzalawy A, et al. CNP deficiency causes severe hypomyelinating leukodystrophy in humans. *Hum Genet*. 2020;139(5):615-622. doi:10.1007/s00439-020-02144-4

37. Sferra A, Fortugno P, Motta M, et al. Biallelic mutations in *RNF220* cause laminopathies featuring leukodystrophy, ataxia and deafness. *Brain*. 2021;144(10):3020-3035. doi:10.1093/brain/awab185

38. Guasto A, Dubail J, Aguilera-Albesa S, et al. Biallelic variants in *SLC35B2* cause a novel chondrodysplasia with hypomyelinating leukodystrophy. *Brain*. 2022;145(10):3711-3722. doi:10.1093/brain/awac110

39. Fuijkschot J, Theelen T, Seyger MMB, et al. Sjögren–Larsson syndrome in clinical practice. *J Inherit Metab Dis*. 2012;35(6):955-962. doi:10.1007/s10545-012-9518-6

40. Haug S, Braun-Falco M. Restoration of fatty aldehyde dehydrogenase deficiency in Sjögren–Larsson syndrome. *Gene Ther*. 2006;13(13):1021-1026. doi:10.1038/sj.gt.3302743

41. Crow YJ, Shetty J, Livingston JH. Treatments in Aicardi–Goutières syndrome. *Dev Med Child Neurol*. 2020;62(1):42-47. doi:10.1111/dmcn.14268

42. Zhang S, Song J, Yang Y, et al. Type I interferonopathies with novel compound heterozygous TREX1 mutations in two siblings with different symptoms responded to tofacitinib. *Pediatr Rheumatol*. 2021;19(1):1. doi:10.1186/s12969-020-00490-1

43. Meesilpavikkai K, Dik WA, Schrijver B, et al. Efficacy of Baricitinib in the Treatment of Chilblains Associated With Aicardi‐Goutières Syndrome, a Type I Interferonopathy. *Arthritis Rheumatol*. 2019;71(5):829-831. doi:10.1002/art.40805

44. Vanderver A, Adang L, Gavazzi F, et al. Janus Kinase Inhibition in the Aicardi–Goutières Syndrome. *N Engl J Med*. 2020;383(10):986-989. doi:10.1056/NEJMc2001362

45. Taşdelen E, Durmaz CD, Karabulut HG. Autosomal Recessive Oculodentodigital Dysplasia: A Case Report and Review of the Literature. *Cytogenet Genome Res*. 2018;154(4):181-186. doi:10.1159/000489000

46. Yu LH, Morimura T, Numata Y, et al. Effect of curcumin in a mouse model of Pelizaeus–Merzbacher disease. *Mol Genet Metab*. 2012;106(1):108-114. doi:10.1016/j.ymgme.2012.02.016

47. Hubbard WC, Moser AB, Liu AC, et al. Newborn screening for X-linked adrenoleukodystrophy (X-ALD): Validation of a combined liquid chromatography–tandem mass spectrometric (LC–MS/MS) method. *Mol Genet Metab*. 2009;97(3):212-220. doi:10.1016/j.ymgme.2009.03.010
